# Restricted nature of adult neural stem cells: re-evaluation of their potential for brain repair

**DOI:** 10.3389/fnins.2014.00162

**Published:** 2014-06-17

**Authors:** Kirsten Obernier, Cheuk Ka Tong, Arturo Alvarez-Buylla

**Affiliations:** Department of Neurological Surgery, The Eli and Edythe Broad Center of Regeneration Medicine and Stem Cell Research, University of California, San FranciscoSan Francisco, CA, USA

**Keywords:** adult neural stem cells, V-SVZ, brain repair, circuit plasticity, specification

## Abstract

Neural stem cells (NSCs) in the walls of the lateral ventricles continue to produce new neurons and oligodendrocytes throughout life. The identification of NSCs, long-range neuronal migration, and the integration of new neurons into fully formed mature neural circuits—all in the juvenile or adult brain—has dramatically changed concepts in neurodevelopment and suggests new strategies for brain repair. Yet, the latter has to be seen in perspective: NSCs in the adult are heterogeneous and highly regionally specified; young neurons derived from these primary progenitors migrate and integrate in specific brain regions. Neurogenesis appears to have a function in brain plasticity rather than brain repair. If similar processes could be induced in regions of the brain that are normally not a target of new neurons, therapeutic neuronal replacement may one day reinstate neural circuit plasticity and possibly repair broken neural circuits.

For many years, it was believed that once development is completed, no new nerve cells are added to the central nervous system. This view imposed a severe limitation on our thinking of possible mechanisms for neuronal replacement and brain repair. This dogma began to change with observations made by Joseph Altman in the 1960's: [H]^3^-thymidine labeled progenitors gave rise to cells in several brain regions that had the morphology of neurons under the light microscope (Altman, 1962). However, the identity of these labeled cells remained highly controversial for many years (see: Rakic, 2002). Conclusive evidence for adult neurogenesis in homeotherms came from the work of Fernando Nottebohm and colleagues in the 1980's using electrophysiology, electron microscopy, and tracer methods. This work in adult songbirds, where seasonal changes in the size of song control nuclei had been previously documented, showed that new neurons continually replace older cells that have died (see: Nottebohm, 2002). Subsequent studies in mammals demonstrated that neurons are continually added to the adult olfactory bulb (OB) and the dentate gyrus during juvenile and adult life (for review see: Ihrie and Alvarez-Buylla, 2011; Gage and Temple, 2013).

New OB neurons are born postnatally within an extensive germinal zone lining the walls of the lateral ventricles. The ventricular-subventricular zone (V-SVZ) is the largest germinal niche in the adult mammalian brain. This germinal layer has been most extensively studied in rodents (Fuentealba et al., 2012; Tong and Alvarez-Buylla, 2014). Primary progenitor cells, which are frequently referred to as NSCs, generate new neurons and oligodendrocytes in the juvenile and adult brain. From the early observations in mammals and songbirds, to the more recent work in the mammalian hippocampus and the V-SVZ, adult neurogenesis has been proclaimed to offer a new hope for brain repair (Nottebohm, 1985; Gage and Temple, 2013). Certainly, the discovery of adult neurogenesis demonstrates that neuronal birth, migration over extremely long distances, and the integration of these cells into established brain circuitry *is indeed all possible*. However, as we discuss below, the NSCs, progenitors, and signaling that allow for new neurons to be born and the mechanisms that permit young neurons to migrate and integrate within the adult brain, do not seem to be intended for brain repair (summarized in Table 1). Instead, most of the evidence suggests that adult neurogenesis enables constant modification of neural circuits—likely related to unique forms of brain plasticity in which some key neurons are eliminated and replaced. Here we focus most of our discussion on the V-SVZ where NSCs, their lineages, and the migration of young neurons have been extensively characterized.

**Table 1:** **Adult Neurogenesis in the V-SVZ: Challenges for Brain Repair**.

**Adult V-SVZ neurogenesis**	**Circuit plasticity**	**Brain repair?**
Cell type specification	• Primary progenitors are regionally specified (Merkle et al., 2007)	• No confirmed evidence for differentiation into cell types required for replacement of lost neurons in different brain regions (Herrera et al., 1999; Raedt et al., 2009)
	• Generation of GABAergic interneurons	
	• Replacement of specific subsets of older neurons in the OB	
Migration of young neurons	• Mostly restricted to the V-SVZ and RMS, directed toward the OB (Lois and Alvarez-Buylla, 1994; Doetsch and Alvarez-Buylla, 1996)	• Requires exiting the V-SVZ and RMS
		• Requires migration and integration into the lesion site
New neuron integration	• Integration into defined circuits	• Requires integration and survival of young neurons in environments that are normally non-neurogenic
	• Specialized synaptic contacts with mitral and tufted projection neurons (Whitman and Greer, 2009)	• Synaptic contacts with different neuronal partners depending on the types of circuit damaged
In context of human brain	• Mostly restricted to infants (Sanai et al., 2004, 2011; Bergmann et al., 2012)	• Limited evidence of neurogenesis under pathological conditions in the adult or aged human brain

In the adult rodent V-SVZ a subpopulation of progenitors with astroglial characteristics, known as B1 cells, function as the NSCs. B1 cells generate young neurons that migrate long distances via the rostral migratory stream (RMS) to the OB where they differentiate into local circuit GABAergic interneurons. For potential brain repair, the understanding of the plasticity of these primary progenitors is key. If these cells are capable of generating a wide diversity of neuronal cell types they could potentially be directed to form nerve cells lost in a disease or following trauma. Earlier work suggested that NSCs could be plastic and that the environment played a key role in directing their differentiation into specific neuronal cell types (Fallon et al., 2000; Shihabuddin et al., 2000). It was suggested that NSCs isolated from one brain region could function as those in another (e.g., hippocampal NSCs transplanted into the V-SVZ) (Brustle and McKay, 1996; Suhonen et al., 1996), whereas more recent work suggests that V-SVZ cells cannot acquire cortical, striatal or hippocampal properties following transplantation (Herrera et al., 1999; Raedt et al., 2009). Additional evidence indicates that NSCs under normal physiological conditions are highly specialized and regionally specified in a cell-autonomous manner to produce specific types of neurons destined for unique circuits within particular brain regions (Merkle et al., 2007). This limitation may be overcome by the trans-differentiation of the precursors using transcription factors (Chen et al., 2012), but in their default state their differentiation seems to be limited.

The specification of the neuroepithelium occurs early in development, restricting the potency of NSCs in the forebrain (Rubenstein, 2011) as well as the spinal cord (Jessell et al., 2011). In the developing murine embryonic brain early progenitors produce striatal, cortical, and septal neurons—possibly orchestrated by combinations of several transcription factors (Rubenstein, 2011)—but later progenitors fail to generate earlier fates (Shen et al., 2006). Whereas depending on their position within the VZ germinal zone embryonic NSCs generate both glutamatergic and GABAergic neurons for different forebrain regions, adult V-SVZ NSCs mostly generate GABAergic neurons destined for the OB. In the adult V-SVZ, NSCs are not only restricted to differentiate into OB interneurons but are heterogeneous and regionally specified (Figure 1). Adenovirus-mediated labeling of NSCs in different locations of the lateral, medial, and pallial wall has shown that dorsal NSCs generate mostly superficial granule cells (GCs) and dopaminergic tyrosine hydroxylase (TH)-positive (but not calbindin-positive) periglomerular cells (PGCs), while ventral NSCs produce deep GCs and calbindin-positive (but not TH-positive) PGCs and NSCs in the medial wall give rise to calretinin-positive PGCs and GCs. This positional specification seems to be primarily cell intrinsic, since labeled progenitors maintained their original phenotype *in vitro* and upon heterotopic grafting within the niche (Merkle et al., 2007). The positional identity of adult NSCs is likely inherited from the early embryonic differential expression of combinations of transcription factors by neuroepithelial and radial glia cells (for review see Kriegstein and Alvarez-Buylla, 2009; Rubenstein, 2011). Furthermore, transplantation of V-SVZ NSCs to non-neurogenic regions (outside the germinal niche) of the adult brain under normal physiological conditions is not permissive for neurogenesis; V-SVZ cells grafted into the cortex or striatum fail to migrate and produce few, if any, neurons (Herrera et al., 1999; Seidenfaden et al., 2006). For now it seems that NSCs and progenitor cells in the V-SVZ are highly restricted in their potential; this may 1 day be overcome by reprogramming.

**Figure 1:**
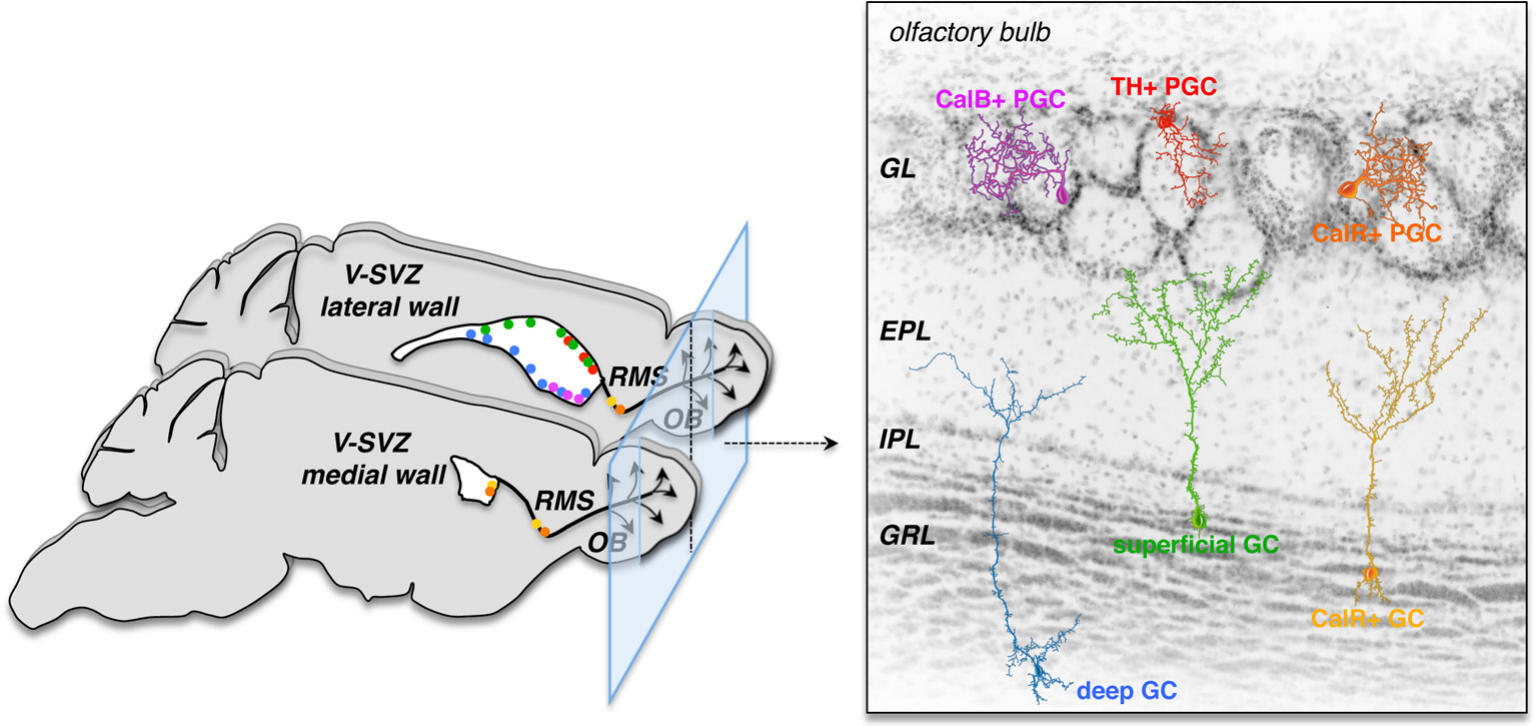
**V-SVZ NSCs are regionally specified and generate unique subtypes of OB interneurons depending on their location**. This specified nature of primary progenitors sets limits to their direct use for brain repair; yet future strategies may exploit reprogramming to induce these NSCs to produce specific neuronal subsets required for brain repair. Left panel: the color dots in the medial (bottom) and lateral (top) walls of the lateral ventricles on the two sagittal views of the left mouse brain depict regions were specific subsets of olfactory bulb (OB) interneuron subtypes (right panel) are born. CalB, Calbindin; CalR, Calretinin; EPL, external plexiform layer; IPL, internal plexiform layer; GC, granule cell; GL, glomerular layer; GRL, granule cell layer; OB, olfactory bulb; PGC, periglomerular cell; RMS, rostral migratory stream; TH, tyrosine hydroxylase; V-SVZ, ventricular-subventricular zone (modified from Alvarez-Buylla et al., 2013).

An important obstacle for brain repair in the juvenile or adult brain is the long distances that frequently separate endogenous germinal niches, or sites of transplantation of progenitor cells, from the sites where new neurons would be required. Damaged areas are also frequently distributed over large volumes of brain tissue. Repair, therefore, requires long migrations of precursor cells through the complex postnatal brain parenchyma. NSCs (B1 and radial glia cells) are largely fixed to specific locations in the neuroepithelium. In contrast, young neurons derived from these specified locations need to migrate to their final destination, often very long distances. Consistently, neuroblasts derived from the adult V-SVZ, have an extraordinary capacity for long-range migration within the adult brain parenchyma (Lois and Alvarez-Buylla, 1994). In the context of brain repair, this migratory capacity could potentially allow young neurons to penetrate deep into a lesion and replace neurons where they are needed. However, under normal conditions, neuroblasts derived from the V-SVZ migrate within distinct corridors within the SVZ and the RMS (Doetsch and Alvarez-Buylla, 1996). This constrained migration mediated by chemotactic factors, glial ensheathment, and a vascular scaffold (Nguyen-Ba-Charvet et al., 2004; Sawamoto et al., 2006; Whitman et al., 2009; Kaneko et al., 2010) ensures the delivery of these young neurons to the OB, where they then disperse radially into specific layers (Whitman and Greer, 2009; Ihrie and Alvarez-Buylla, 2011). Interestingly, there is some evidence that neuroblasts are able to leave the V-SVZ and RMS to invade the adjacent striatum. For example, following ischemia-induced activation of guidance molecules (e.g., CXCR4-SDF1) neuroblasts migrate toward the ischemic site but the ectopic migration may be due to aberrant expression of substrate and guidance molecules within the context of the lesions. It is also possible that the physical damage from these lesions could interfere with the normal migratory path, facilitating the derailment of neuroblasts into adjoining territories while they maintain their intrinsic differentiation potential and thus are rather misdirected OB interneurons (Liu et al., 2009). Although it has been suggested that few young neurons migrating toward the site of lesion can survive and possibly integrate (Arvidsson et al., 2002; Kokaia et al., 2006; Yamashita et al., 2006; Hou et al., 2008), most do not become mature neurons and do not survive long-term (Osman et al., 2011; Cui et al., 2013). Others have reported that intraventricular growth factor infusion may be necessary to promote regeneration (Kolb et al., 2007). In contrast to neurons, there is better evidence that oligodendrocyte progenitors derived from the V-SVZ can migrate into a demyelinated lesioned site and differentiate into new oligodendrocytes (Nait-Oumesmar et al., 1999; Picard-Riera et al., 2002; Menn et al., 2006). Upon brain injury some V-SVZ cells may also produce astrocytes that migrate to the injury site (Benner et al., 2013). Therefore, migration through the adult brain is limited to very specific paths and to specific subtypes of neurons and glial cells.

The ability to become synaptically incorporated into fully formed adult brain circuits is an essential characteristic of new neurons formed in the V-SVZ. While synaptic incorporation is a highly desired property for putative new neurons in the context of brain repair, these cells specifically integrate into the circuits of the OB. In the OB they mediate inhibition of mitral and tufted projection neurons and it is thought that their continual replacement into adulthood is associated to olfactory discrimination and plasticity (Gheusi et al., 2000; Cecchi et al., 2001; Sakamoto et al., 2011). Plasticity-related functions for adult-born neurons have also been proposed in songbirds (Alvarez-Buylla et al., 1990; Scharff et al., 2000) and in the rodent hippocampus (Kempermann, 2012; Gage and Temple, 2013). Therefore, the available evidence strongly suggests that the integration of newly formed neurons occurs within very specific circuits where ongoing plasticity requires new nerve cells. It is tempting to speculate that diseased or injured brain circuits could similarly present a permissive environment for the recruitment of new neurons, but this remains to be demonstrated. If the young neurons that are normally produced in the adult brain are so highly tuned to specific brain circuits, an important limitation might be their competence to differentiate into neuronal cell types that can migrate and integrate to reconstitute function in damaged circuits. Neuronal progenitors capable of differentiation, migration and integration within the environment of neurodegeneration or trauma may exist, but these cells do not seem to be the ones normally produced in the adult brain.

The above shows that the birth, migration, and integration of young neurons is fashioned for specific brain circuits with the demand for a special form of plasticity in the adult brain. This sets limits to the use of adult NSCs in brain repair. Species-specific differences in adult neurogenesis may also determine the potential use of NSCs in brain repair. For example, it has been known for sometime, that reptiles and amphibians can regenerate neuronal populations that are not repaired in mammals or birds (Polenov and Chetverukhin, 1993; Font et al., 2001; Garcia-Verdugo et al., 2002; Chapouton et al., 2007). However, there are also important differences among mammals. The human V-SVZ differs in its cytoarchitecture compared to the rodent germinal zone. While the human V-SVZ is also lined with a monolayer of ependymal cells at the apical side of the walls of the lateral ventricles, it basally consists of a hypocellular gap, an astrocytic ribbon that contains neural progenitor cells, and a transition zone into the parenchyma. In infant humans the V-SVZ is an important source of new neurons not only for the OB, but also for specific subregions of the anterior prefrontal cortex (Sanai et al., 2004, 2011; Yang et al., 2011). Unlike in rodents where neurogenesis persists throughout the animal's lifespan—though it is drastically reduced during aging—only very few migrating young neurons are observed in the adolescents or in adult human brains. Consistently, radiocarbon birth dating suggests that the vast majority of neurons in the OB are as old as the person, implying they are born during early development (Bergmann et al., 2012). Those neurons that are added to specific brain regions during early childhood could be key in understanding critical-period plasticity and postnatal developmental deficits. In the hippocampus, the story may be somewhat different when compared to the OB. It remains controversial how many new neurons continue to be added in the dentate gyrus in the adult human brain. Some studies suggest the presence of newly born hippocampal neurons in adults and a surprisingly stable birth rate from adolescence to aging (Eriksson et al., 1998; Spalding et al., 2013), yet other studies reveal very few cells expressing markers of young neurons in the hippocampus after birth (Knoth et al., 2010). In adult humans, in addition to the intrinsic limitations imposed by adult NSC specification, the possible low number of NSCs or their long-term quiescence pose further constraints, which may limit their potential use in cell replacement therapies. Even if the activation of resident quiescent NSCs—if they exist—is possible, the migration, survival, and integration into specific brain regions may be a greater challenge given the large size of the human brain.

While we have made significant progress in understanding the identity and lineages of adult NSCs, there is no evidence that these cells can repair neural circuits or replace diverse neuronal cell types. The function of adult neurogenesis remains unknown, but significant evidence points to processes of brain plasticity rather than brain repair. Regions of the postnatal brain that continue to receive new neurons may be plastic throughout life; they may not be constrained by defined critical-periods of plasticity that ends as their neuronal components mature (Southwell et al., 2010). The addition of new neurons may be seen as a constant infusion of youth into mature neural circuits. The more directed use of neuronal replacement for brain repair will likely require very specific types of progenitor cells that can navigate and appropriately integrate into target regions. Most likely this will involve the reprograming of embryonic or adult brain cells for the production of those specific neuronal cell types that can integrate and repair damaged neural circuits. Neural progenitor cells from the mouse embryonic MGE for example have been shown to migrate long distances upon transplantation and can contribute to neuronal replacement, although only with the generation of interneurons, not excitatory neurons (Wichterle et al., 1999; Alvarez-Dolado et al., 2006; Southwell et al., 2010). Human fetal cells transplanted into stroke-lesioned mice migrate long distances and differentiated into mature neurons in the lesioned striatum or cortex (Kelly et al., 2004; Darsalia et al., 2007). Also further efforts in the field of iPSC-derived neural progenitors may lead to neuronal replacement strategies. We also cannot exclude that very specific subsets of the heterogeneous population of endogenous adult V-SVZ NSCs may harbor the potential to function as precursors for neurons in different brain regions; this requires detailed analysis of the potential of individual NSCs located in different regions of the adult V-SVZ.

The identification of NSCs among astroglia and the abundance of glial cells suggest that the adult brain may contain a large reservoir of cells that could be target for such reprograming. Efforts along these lines have already begun (Guo et al., 2013; Niu et al., 2013), but further understanding of astrocyte heterogeneity is required (Hochstim et al., 2008; Tsai et al., 2012).

In the present perspective article we have focused mostly on adult neurogenesis in the V-SVZ, as this is the most extensive germinal region of the adult mammalian brain. Similar concepts of specification, confined migration, and integration probably also apply to neurogenesis in the adult hippocampus and avian forebrain (Scharff, 2000; Nottebohm and Liu, 2010; Ming and Song, 2011; Gage and Temple, 2013). This very high level of specification may suggest, to some, that adult neurogenesis is irrelevant to brain repair. However, this is not the case: one important lesson learned from studies of adult neurogenesis is that processes considered impossible three decades ago do indeed take place in the adult brain; young neurons can be produced and they can migrate, and most importantly integrate, into adult brain circuits. Their seamless migration and integration, whether in the song control circuits of birds, or in the OB and hippocampus of adult mammals, still has much to teach us about how to accomplish similar neuronal replacement for neurons lost during neurodegeneration or trauma.

## Conflict of interest statement

The authors declare that the research was conducted in the absence of any commercial or financial relationships that could be construed as a potential conflict of interest.
